# A two-compartment upflow pilot scale bioreactor system for microbial sulfate reduction control studies

**DOI:** 10.1016/j.mex.2019.02.020

**Published:** 2019-02-26

**Authors:** L.R.P. de Andrade Lima, L.A. Bernardez

**Affiliations:** Department of Materials Science and Technology, Federal University of Bahia, Brazil

**Keywords:** Bioreactor, Sulfate reducing bacteria, Souring

## Abstract

Souring in oil fields occurs mainly due to the activity of sulfate reducing bacteria (SRB). Most of the studies on SRB are performed using upflow packed-bed reactors that have a limitation to describe the region close to the injection wells in oil fields, which is characterized by void and saturated porous bed regions. Here, it is described the design and operation of a pilot scale system to investigate the SRB activity, inhibition and control in oil fields.

• The bioreactor is composed by two-compartments (empty and packed-bed).
• The reactor system has two parallel bioreactors that can be supplied with the same source of nutrients through a single pump or can be supplied separately with different solutions using two pumps.
• The hydrodynamics for conventional packed bed bioreactors has a mixing behavior dependent of the flow rate and has a significant by-pass. In contrast, the two-compartment system presented here has a mixing behavior almost independent of the flow rate.

## Graphical abstract

**Figure:**
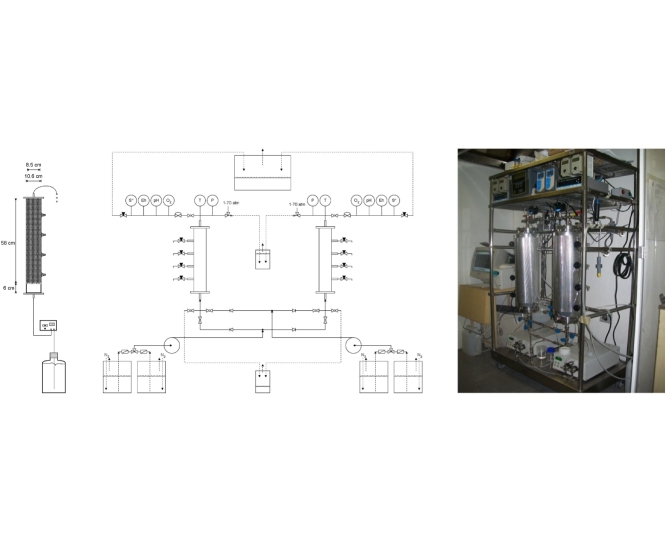

**Specifications Table**

**Table:** 

**Subject Area:**	*Environmental Science*
**More specific subject area:**	*Environmental Microbiology*
**Method name:**	SRB Bioreactor System
**Name and reference of original method:**	*not applicable*
**Resource availability:**	*not applicable*

## Method detail

Souring in oil fields occurs mainly due to the activity of sulfate reducing bacteria (SRB). These microorganisms remain in the surrounding environment near the injection wells. The souring inhibition and control is an active research area, because small scale experiments are essential to define reactants limit concentrations and injection strategies to control the microorganism population and the sulfide generation. Despite the fact that most of the studies on SRB are performed using upflow packed-bed reactors [[1], [2], [3], [4], [5], [6], [7]], these reactors do not reasonably describe the regions close to the injection wells, which are characterized by the nearness of a saturated porous bed and void regions due to the injection wells.

### System description

**Fig. 1:**
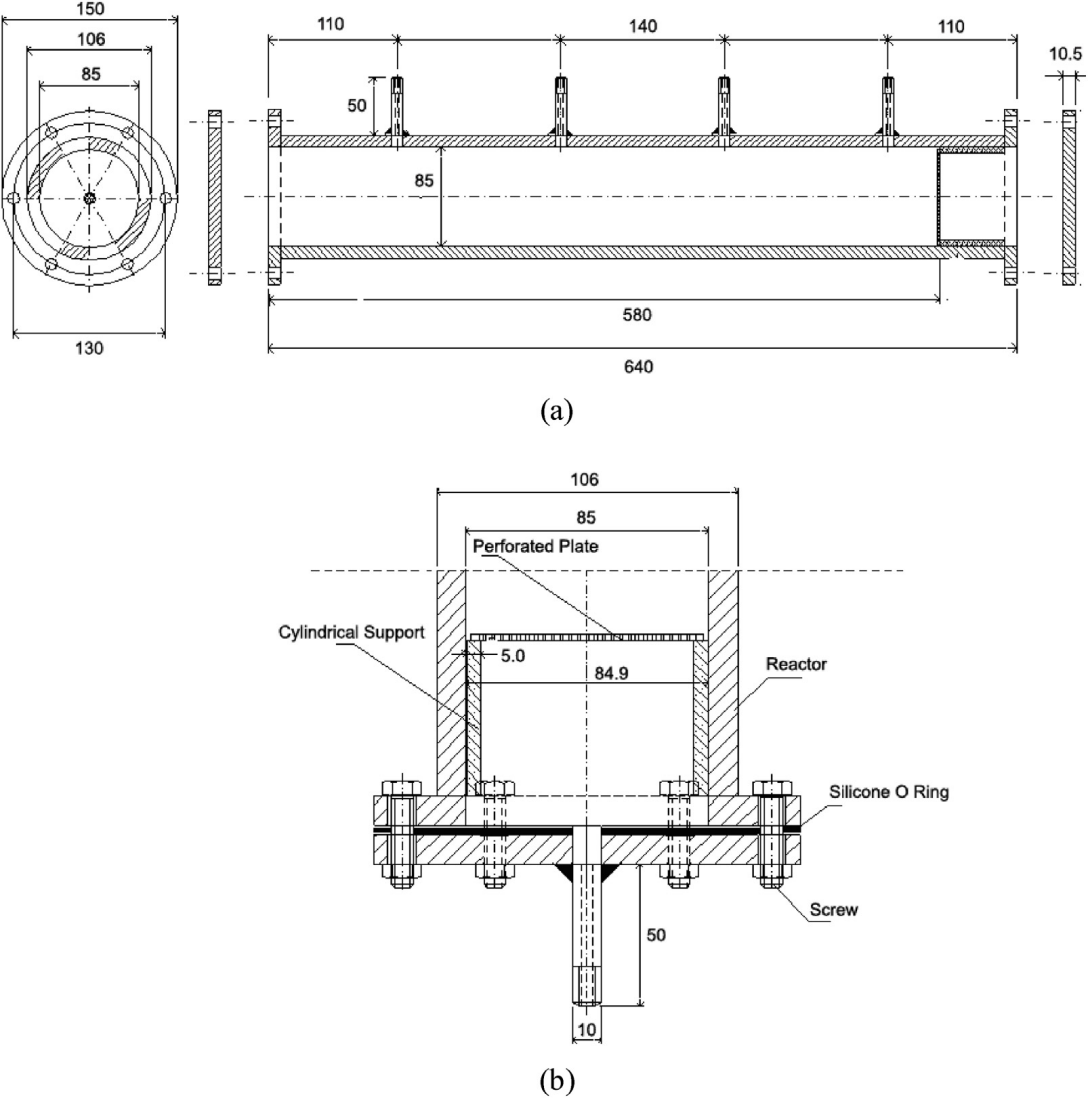
(a) Bioreactor longitudinal section, b) constructive details of the bioreactor bottom.

The system presented is a pilot-scale unit composed by two-compartment upflow packed-bed bioreactors. The bioreactors are cylindrical-shaped devices with a work volume of 3.6 L, filled with a porous medium to support microbial growth. The material of the bioreactor is the super duplex stainless steel UMS S32750 (SAF 2507, Sandvik Steel) in order to avoid corrosion due to presence of hydrogen sulfide gas, hydrogen, aqueous solution rich in chloride ions and temperatures about 70 °C. The details of the unit are shown in Fig. 1a and b and are composed of the main parts described below: a) cylindrical body in flanged superduplex stainless steel containing four sampling points; b) length of the tube equal to 64 cm; c) internal and external diameter, respectively, equal to 85 and 106 mm; d) flanged top and base with bolted caps with 1 1/2" (3.81 mm) × 10 mm hexagonal screws; e) a point of entry and exit of the effluent at the base and top of the bioreactor respectively; f) circular bushing in super duplex stainless steel to support perforated plates in super duplex stainless steel with holes of 1 or 2 mm in order to maintain the porous medium; g) the distance between the sampling ports is 14 cm with a length of 50 mm and a diameter of 10 mm; h) the caps, cylindrical body with flanges, four side tubes and the two at the ends were carefully machined from a super duplex stainless steel rod; i) the six tubes were then carefully welded onto the reactor body and the lids.

**Fig. 2:**
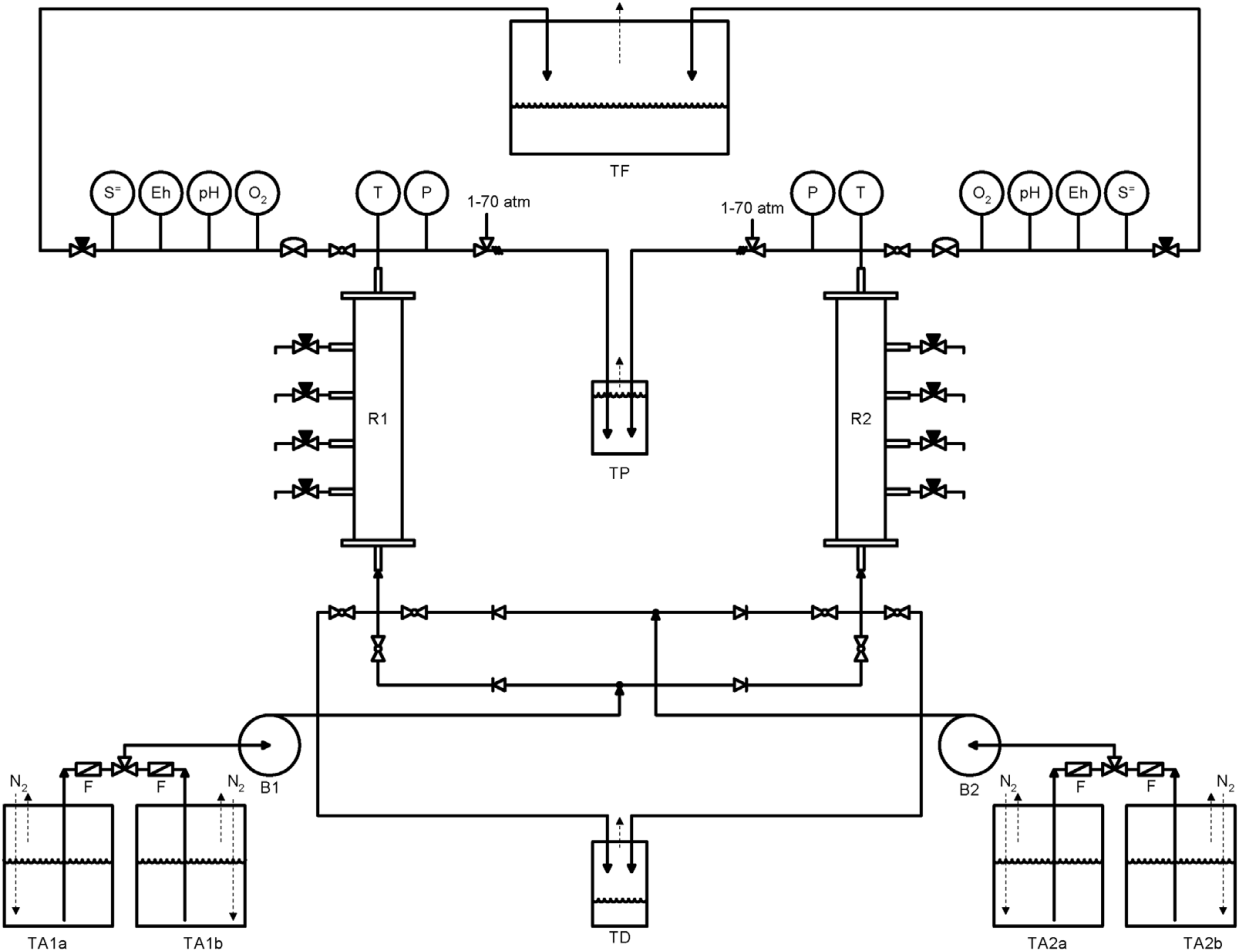
Bioreactors system assemblage.

**Fig. 3:**
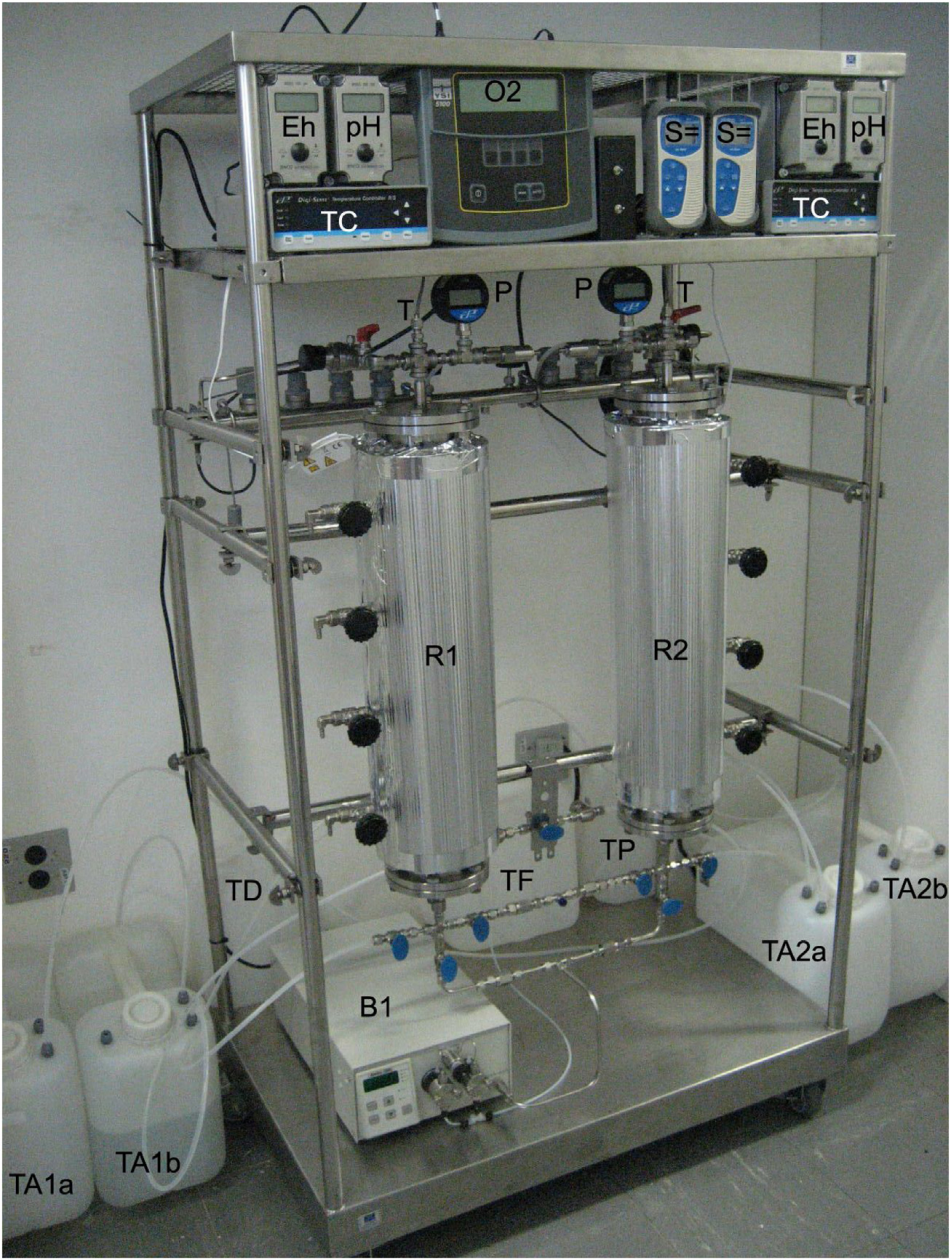
Bioreactor system.

The pilot plant (described in Fig. 2, Fig. 3) contains two packed upflow packed bed reactors (R1 and R2) for the anaerobic sulfate reduction process, including a produced gas collection system (TF and TP), which contains a salt which generates a sparingly soluble sulfide. The bioreactor allows continuous tests at atmospheric pressure and under pressure of up to 70 atm (7093 kPa) at temperatures up to 70 °C.

### System operation

From two feed tanks (TA1a and TA1b for reactor R1 and TA2a and TA2b for reactor R2) the source of sulfate and other nutrients is filtered (F) and transported by a constant flow high-pressure pumps (B1 and B2) and circulated through the column through the point of entry. The solution percolates the porous matrix, composed of glass beads, sand or core to support the growth of the sulfate reducing bacteria, injected at each sampling point. The hydrogen sulfide produced is directed to the top of the reactor and transferred to reservoirs containing a trap solution. Samples can be taken manually at each sampling point for analysis of bacterial activity through the concentrations of sulfate, hydrogen sulfide, proteins and organic acids. The unit pipes and valves are made in AISI 316 stainless steel. It was included a drainage system to release the aqueous solution to a tank with NaOH (TD) for the case of H_2_S emission. To ensure anaerobic environment nitrogen is injected into the feed tanks through pipes connected to a gas cylinder.

The pilot unit is instrumented (described in Figs. 2 and 3) with sensors of pressure (P), temperature (T), dissolved oxygen (O_2_), pH, redox potential (Eh) and sulfide (S^=^). The bioreactor is heating by passing electric current through an isolated and grounded heating tape; the temperature controller (TC) sets the electrical current to minimize the temperature set-point. Fig. 3 shows details of the actual system.

The pilot-scale unit described is multi-purpose: 1) From a feed tank the medium with nutrients can be pumped by an constant flow high pressure pump and circulated by the two columns, one of which works as a test column with the presence of bacteria and another as a control without bacteria. The growth kinetics of SRB and its products can then evaluated; 2) A bioreactor containing the SRB can fed with solution with a sulfate source and the other nutrients through the pump while the second pump circulates another nutrient source in the second bioreactor to evaluate the effect on the kinetics of the sulfate reducing bacteria; 3) Bacteria other than sulfate reducing bacteria can be inoculated in the second reactor as is the case of native microorganisms to evaluate competition with SRB and the result compared to the bioreactor containing only SRB.

### Method validation

**Fig. 4:**
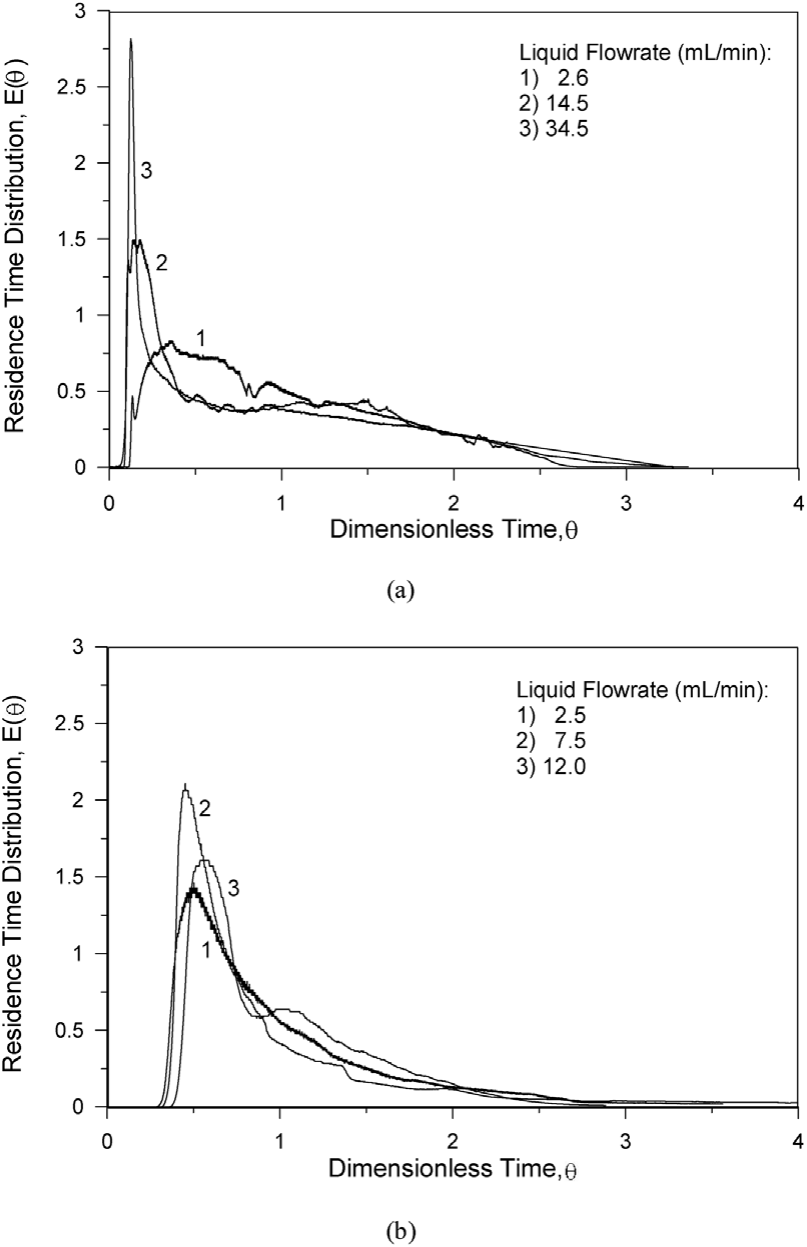
Residence time distribution at low and intermediate flow rate conditions for packed bed bioreactor: a) conventional one compartment reactor, b) two-compartments reactor.

Previous studies on SRB kinetics [[1], [2], [3], [4], [5], [6], [7]] using batch and continuous bioreactors demonstrate the feasibility of laboratory scale studies to evaluate the action of specific bacteria communities on the sulfate conversion to sulfide. The hydrodynamics of the conventional continuous packed bed upflow bioreactor [6] and the present two-compartment upflow bioreactor [8,9] were addressed. Fig. 4a shows the residence time distribution at low and intermediate flow rate conditions for a conventional one compartment upflow packed bed bioreactor and Fig. 4b shows the residence time distribution for the two-compartment upflow packed bed bioreactor. One remarks that the conventional system has a mixing behavior very dependent of the flow rate and has a significant by-pass. At low velocities the behavior has a continuous stirred tank reactor component and at high velocity the plug flow behavior is dominant. The two-compartment system has a mixing behavior analogous for low and intermediate flow rates.
